# The Margret M. and Paul B. Baltes Award Lecture and Presentation

**DOI:** 10.1093/geroni/igaf122.809

**Published:** 2025-12-31

**Authors:** Jennifer Ailshire

## Abstract

The Margret M. and Paul B. Baltes Award Lecture will feature an address by the 2024 Baltes Award recipient, Nicole E. Werner, PhD, of Vanderbilt University. This session will also include the presentation of the 2025 Margret M. and Paul B. Baltes Award to recipient Jeremy M. Hamm, PhD, of North Dakota State University. The Margret M. and Paul B. Baltes Foundation Award in Behavioral and Social Gerontology recognizes outstanding early-career contributions in behavioral and social gerontology. The award is generously funded by the Margret M. and Paul B. Baltes Foundation.

